# Transverse colonic volvulus due to mesenteric fibromatosis: a case report

**DOI:** 10.1186/s12876-020-01592-6

**Published:** 2021-01-06

**Authors:** Akihiro Yoshida, Yasutake Uchima, Naoki Hosaka, Kosuke Minaga, Masatoshi Kudo

**Affiliations:** 1 Department of Gastroenterology, Fuchu Hospital, Izumi, Japan; 2 Department of Gastroenterology and Hepatology, Kindai University Faculty of Medicine, Osaka-Sayama, Osaka 589-8511 Japan; 3 Department of Surgery, Fuchu Hospital, Izumi, Japan; 4 Department of Pathology, Fuchu Hospital, Izumi, Japan

**Keywords:** Mesenteric fibromatosis, Transverse colonic volvulus, Desmoid tumor, Right hemicolectomy

## Abstract

**Background:**

Colonic volvulus, a condition in which a colonic segment partially twists around its base, is the third leading cause of large bowel obstruction after colonic neoplasms and diverticular disease. However, volvulus of the transverse colon is the rarest type of large intestinal volvulus. Moreover, the occurrence of transverse colonic volvulus secondary to a benign tumor originating from outside the intestine has never been reported. We hereby report a case of transverse colonic volvulus caused by mesenteric fibromatosis.

**Case presentation:**

A 53-year-old female with a history of rheumatoid arthritis and thyroid tumor presented with abdominal pain for 1 day. Abdominal computed tomography revealed intestinal torsion at the hepatic flexure. Twisted and obstructed mucosa of the transverse colon was observed during colonoscopy, but no tumor invasion of the mucosal surface was detected. A solid mass of a mesenteric origin with involvement of the transverse colon was observed during surgery. The mass was diagnosed surgically as transverse colonic volvulus induced by a mesenteric tumor. Hence, the patient underwent a right hemicolectomy. Histopathological results indicated mesenteric desmoid-type fibromatosis. The postoperative recovery was uneventful, and the patient was discharged 8 days after surgery.

**Conclusions:**

Although mesenteric fibromatosis is rare, this disease should be considered when managing transverse colonic volvulus resulting from nonmucosal tumors.

## Background

Colonic volvulus is a rotation or twisting of the large intestine around its vascular pedicle [1]. According to case descriptions in the literature, it most commonly involves the sigmoid colon (60–70%), followed by the cecum (25–40%), and the transverse colon (1–4%) [2]. Thus, volvulus is rarely observed in the transverse colon compared to other colonic segments. A major cause of colonic volvulus is the presence of a redundant mobile colon with a narrow mesenteric root base [3]. Other predisposing factors for colonic volvulus include a high-fiber diet, constipation, previous abdominal surgery, colonic neoplasm, pregnancy, and neurological and psychiatric diseases [2–4]. Reports of intestinal obstruction due to mesenteric fibromatosis (MF) are rare [5, 6], and no cases of transverse colonic volvulus due to MF have been reported. We herein report a case of transverse colonic volvulus secondary to MF in a 53-year-old female.

## Case presentation

**Fig. 1:**
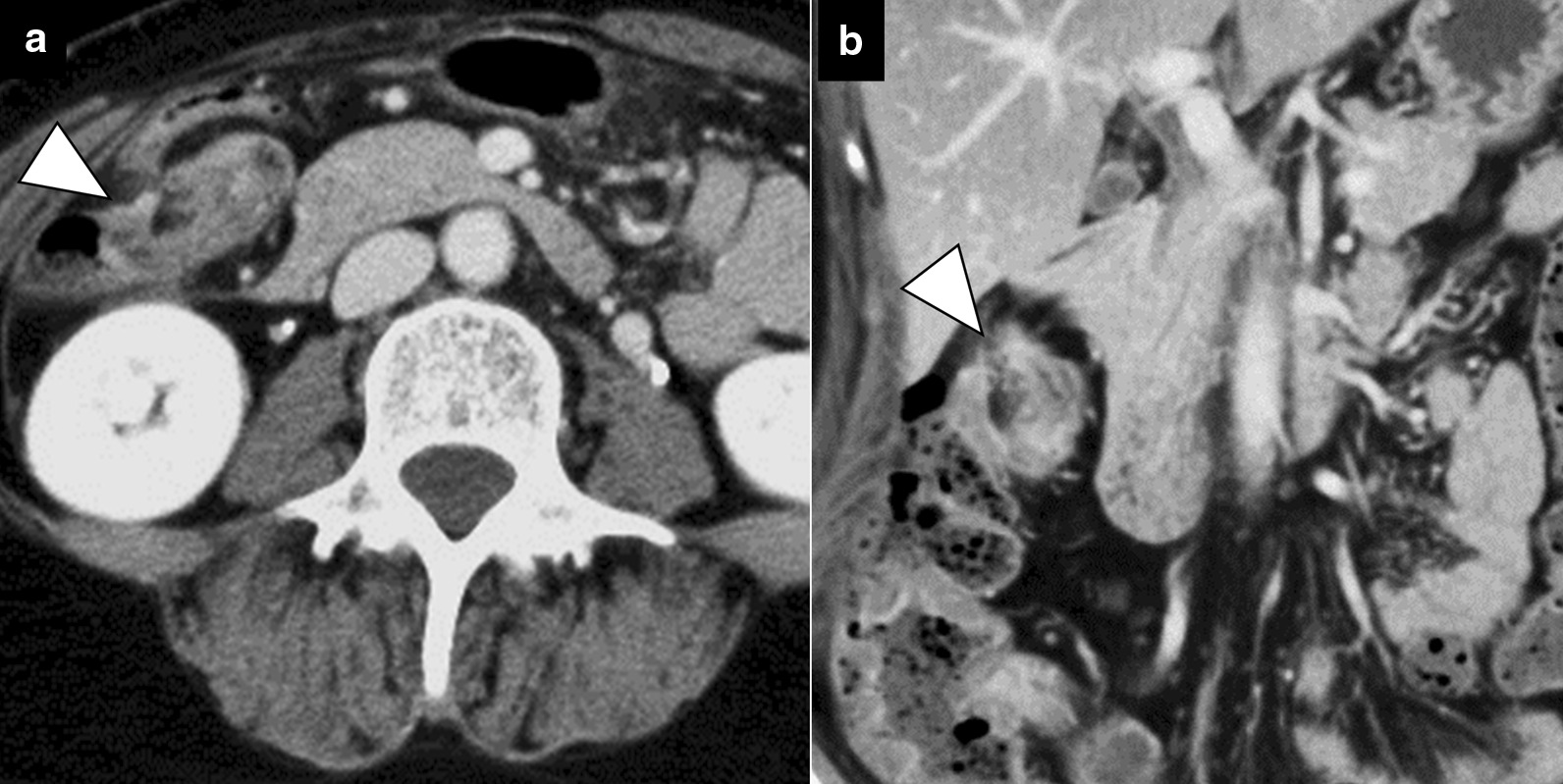
Contrast-enhanced computed tomography showing intestinal torsion at the hepatic flexure (arrowheads) (**a**; axial image, **b**; coronal image)

**Fig. 2:**
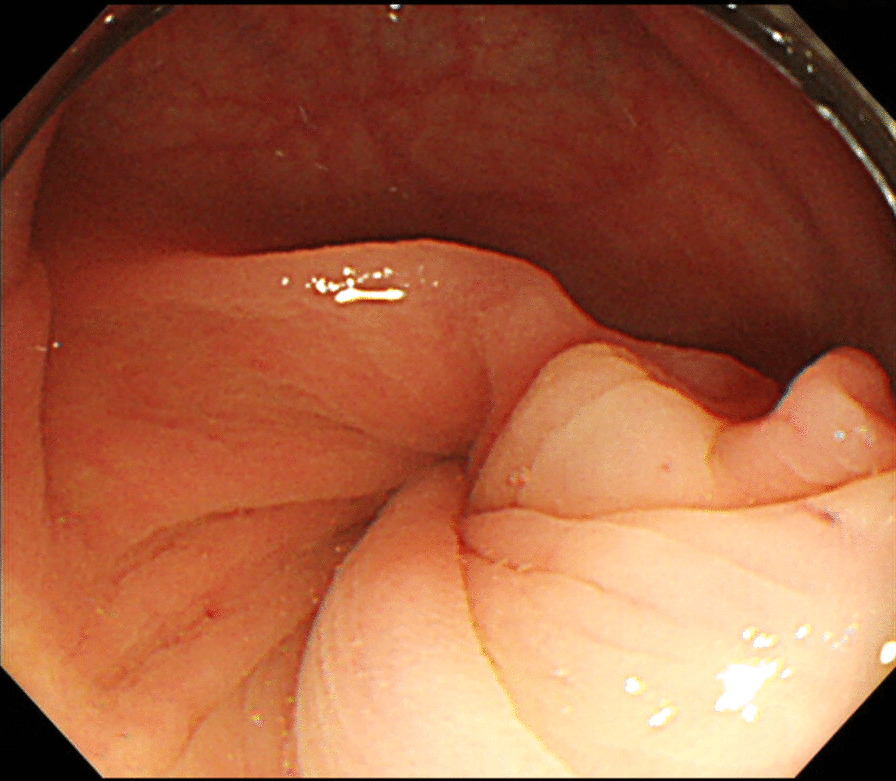
Colonoscopy showing twisted mucosal folds in the transverse colon. No obvious neoplastic change was noted on the mucosal surface

A 53-year-old female with a history of rheumatoid arthritis and thyroid tumor was referred to our hospital. The patient’s chief complaint was abdominal pain for 1 day, and she had no history of abdominal surgery or trauma. Upon admission, she had right abdominal tenderness. Blood tests showed unremarkable results (white blood cell count, 4800/μL; red blood cell count, 4.78 × 10^6^/μL; hemoglobin, 14.8 g/dL; hematocrit, 43.7%; platelets, 20.7 × 10^4^/μL; C-reactive protein, 0.59 mg/dL; creatine kinase, 50 U/L; carcinoembryonic antigen, 2.0 ng/mL; and carbohydrate antigen 19–9, 12 U/mL). Contrast-enhanced computed tomography (CT) revealed intestinal torsion at the hepatic flexure (Fig. 1). A colonoscopy was performed to evaluate the cause of the intestinal torsion, which revealed twisted mucosal folds in the transverse colon but no obvious neoplastic change on the mucosal surface (Fig. 2). The scope could not be advanced past the torsion of the transverse colon, making it impossible to observe the superior colon.

**Fig. 3:**
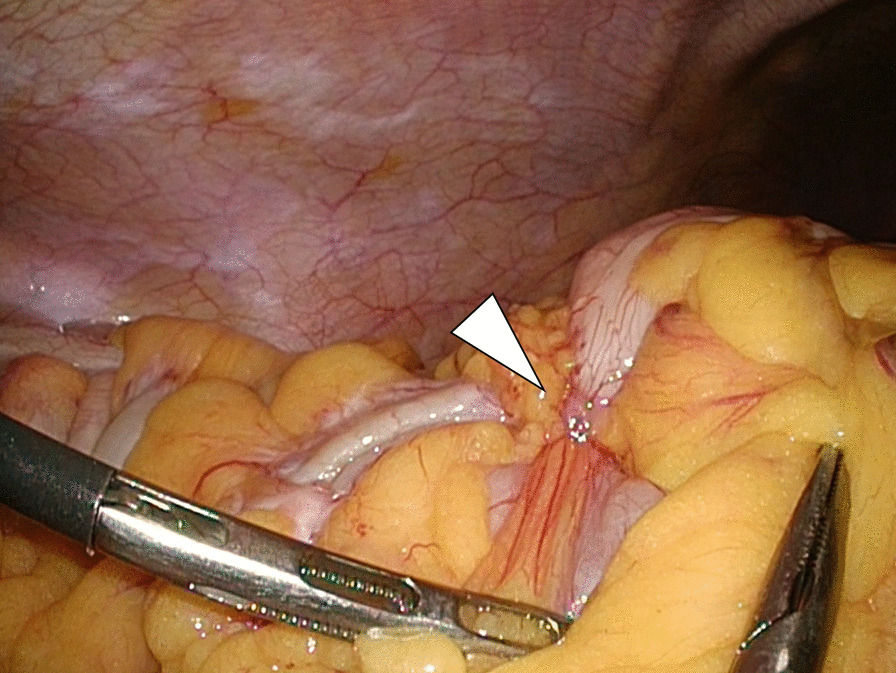
Surgical finding. The tumor was intraoperatively found to have arisen from the mesentery, causing a rotation of the transverse colon around itself (arrowhead)

Based on these findings, the differential diagnosis included gastrointestinal stromal tumor, malignant lymphoma, sarcoma, carcinoid, and mesenteric tumors. Surgery was performed to release the transverse colonic volvulus and establish a definitive diagnosis. The lesion arose from the mesentery, causing a 180° clockwise rotation of the transverse colon around itself (Fig. 3). The surgical diagnosis was transverse colonic volvulus due to a tumor of a mesenteric origin. Consequently, a right hemicolectomy with lymphadenectomy was performed.

**Fig. 4:**
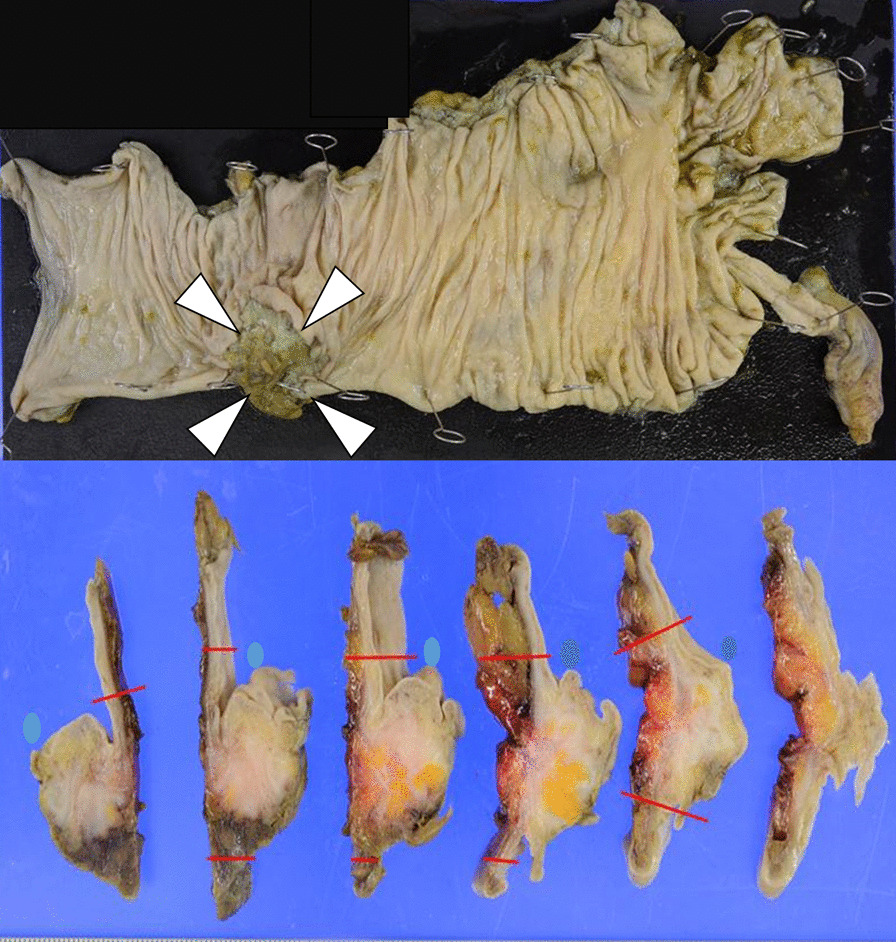
Macroscopic findings. The tumor was a clearly demarcated solid mass with a white–gray appearance

**Fig. 5:**
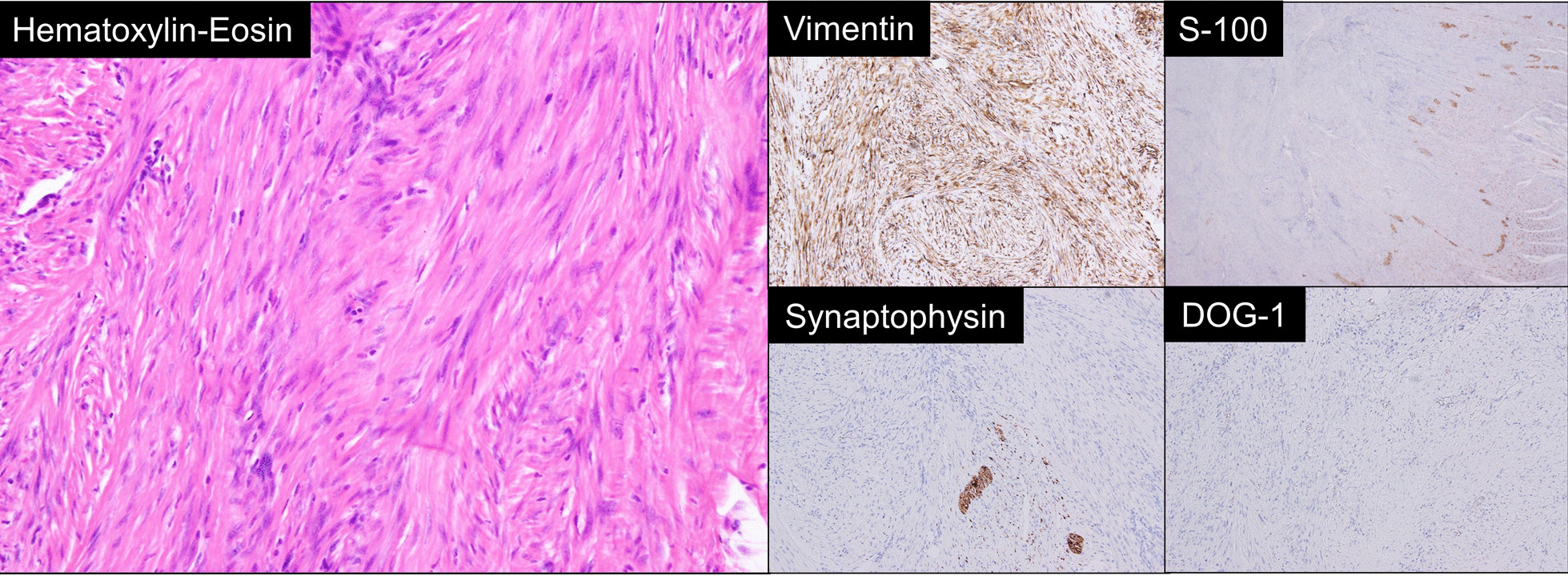
Microscopic findings. Hematoxylin and eosin stain; the tumor consisted of dense collagenous fibers and a complicated proliferation of spindle-shaped cells, which generally appeared heterozygous and homogenous, yet occasionally exhibited nuclear enlargement and mismatch in the center of the tumor. Immunohistochemical stain; the tumor cells were positive for vimentin but negative for S100 protein, synaptophysin, and DOG-1

Macroscopically, the tumor was a clearly demarcated solid mass with a white–gray appearance (Fig. 4). Histologically, the tumor consisted of dense collagenous fibers and a complicated proliferation of spindle-shaped cells, which generally appeared heterozygous and homogeneous, yet occasionally exhibited nuclear enlargement and mismatch in the center of the tumor (Fig. 5). The tumor cells were positive for vimentin but negative for S100 protein, synaptophysin, cluster of differentiation 34, c-kit, and DOG-1, based on the immunohistochemical findings (Fig. 5). These pathological findings led us to the final diagnosis of MF. The patient had an uneventful postoperative course and was eventually discharged in good condition 8 days after surgery. She is currently well and shows no signs of recurrence 6 months after the surgery.

## Discussion and conclusions

MF, also referred to as desmoid fibromatosis, was first described by Muller in 1838 [7]. The annual incidence of MF is only 2–4 per million, accounting for 3% of all soft tissue tumors [8]. MF originates from the mesenchymal tissue and is a type of intra-abdominal fibromatosis, comprising nearly 8% of all fibromatoses. MF has a low morbidity rate [8, 9]. The etiology of MF includes trauma, surgery, hormones, and heredity [7, 9–11]. Furthermore, MF is associated with familial adenomatous polyposis and is a component of Gardner’s syndrome [12, 13]. Fibromatoses can occur almost anywhere in the body and are classified according to their anatomic locations into the following three main categories: extra-abdominal (trunk and extremities), along the abdominal wall, and, least commonly, intra-abdominal. The mesentery is the most probable location for an intra-abdominal desmoid tumor. MF is a fibroblastic growth of the mesentery that occurs either spontaneously or as a result of surgical trauma. MF develops most frequently in the intestinal mesentery, followed by the omentum and mesocolon [14].

The features of intra-abdominal desmoid tumors shown by CT and magnetic resonance imaging (MRI) are related to their histological characteristics and vascularity. Although the CT findings are nonspecific, intra-abdominal desmoid tumors typically appear as well-delineated solid soft tissue masses without calcifications [15]. The signal intensity of MF on MRI reflects the proportion of collagen fibers, spindle cells, and extracellular matrix present [16]. MF most commonly presents heterogeneously with hypo-/hyperintense signals on T2 images and iso-/hypointense signals on T1 images [16, 17]. The hypointense non-enhancing linear bands, presumably representing dense collagen stroma, are characteristic findings on all MRI sequences [17]. For MF diagnosis, the value of diffusion-weighted MRI sequences is unclear [17, 18].

MF is pathologically characterized by a well-differentiated proliferation of fibroblasts, the presence of intercellular collagen fibers, poor cell heteromorphism, the absence of the nuclear fission image, invasive development, and no distant metastasis but local recurrence [19]. Although not pathognomonic of desmoid tumors, spindle cells are common histopathological findings. The immunohistochemical characteristics of mesenteric desmoid tumors include positive staining for vimentin and β-catenin but negative staining for smooth muscle actin, S100, CD117, and CD34 [20].

For symptomatic tumors or tumors that impair function, decisions on the form of therapy should be made after careful consideration of the tumor location and the potential morbidity of the therapeutic option. Surgical resection with negative margins has traditionally been regarded as the mainstay of therapy for abdominal wall and intra-abdominal MF. Nevertheless, recent insights into the natural history of MF have led to a paradigm shift from margin-negative resection to the acceptance of microscopically positive resection or observation with surgery used more selectively [21]. Other treatment options include radiation therapy and systemic medication therapy with the following choices: tamoxifen, which is believed to induce growth suppression in desmoid tumors through interaction with estrogen receptor beta on tumor cells; nonsteroidal anti-inflammatory drugs, such as sulindac; and doxorubicin and methotrexate, with vinca alkaloid-based chemotherapy [22]. MF has a local recurrence rate of 22.2% following curative resection [23]. Thus, clinically and radiographic follow-up of patients should be performed biannually for at least 3 years and then annually [24].

To the best of our knowledge, this is the first report of a case of intestinal volvulus secondary to MF. The current study underscores the fact that MF should be considered as a possible cause of intestinal volvulus. In conclusion, although observed seldomly in clinical practice, MF may lead to intestinal volvulus. Therefore, MF should be considered as a potential cause when encountering cases of transverse colonic volvulus with no apparent tumor derived from the intestinal mucosa.

## Data Availability

Data sharing does not apply to this article because no datasets were generated or analyzed during the current study.

## Abbreviations

CT: Computed tomography
MF: Mesenteric fibromatosis
